# Jian-Pi-Yi-Shen Formula Ameliorates Oxidative Stress, Inflammation, and Apoptosis by Activating the Nrf2 Signaling in 5/6 Nephrectomized Rats

**DOI:** 10.3389/fphar.2021.630210

**Published:** 2021-03-25

**Authors:** Fanyuan Zhou, Xiaohu Zou, Jing Zhang, Ziwei Wang, Yajun Yang, Dongtao Wang

**Affiliations:** ^1^ Department of Traditional Chinese Medicine, Shenzhen Hospital, Southern Medical University, Shenzhen, China; ^2^ Department of Pharmacology, Guangdong Key Laboratory for R&D of Natural Drug, Guangdong Medical University, Zhanjiang, China; ^3^ School of Chinese Medicine, Southern Medical University, Shenzhen, China; ^4^ Department of the Ministry of Science and Technology, Guangxi International Zhuang Medicine Hospital, Nanning, China; ^5^ Department of Nephrology, Shenzhen Traditional Chinese Medicine Hospital, Guangzhou University of Traditional Chinese Medicine, Shenzhen, China

**Keywords:** chronic kidney disease, Jian-Pi-Yi-Shen formula, Nuclear factor-erythroid 2-related factor 2, oxidative stress, inflammation, apoptosis

## Abstract

Chronic kidney disease (CKD) is an increasing global public health problem, with high morbidity and mortality. Jian-Pi-Yi-Shen (JPYS) formula is a representative traditional Chinese medicine formula in the treatment of CKD, which is widely used in clinical practice in China. However, the underlying mechanism has not been well elucidated. In the present study, we measured the markers of apoptosis, inflammation, oxidative stress, and nuclear factor erythroid 2–related factor 2 (Nrf2) signaling to investigate the effects of JPYS formula on renal function and fibrosis and its molecular mechanism in an established animal model of 5/6 nephrectomized (5/6Nx) rats. The results demonstrated that the JPYS formula exerted a significant preventive effect on renal dysfunction and fibrosis, based on analysis of correlative parameters such as urinary protein, SCr, BUN, glomerular sclerosis index, and tubulointerstitial fibrosis score and renal histopathology and ultrastructural pathology of CKD rats. JPYS formula also induced downregulation of gene expression associated with fibrosis, such as TGF-β and type I, III, and IV collagen. Moreover, the JPYS formula showed a significant protective effect in suppressing cell apoptosis according to the results of apoptotic indexes, including increased gene expression of Bcl-2, decreased gene expression of Bax, caspase 3, caspase 9, and the number of TUNEL-positive cells. JPYS formula also ameliorated the activation of the NF-κB-mediated inflammatory pathway, as manifested by the downregulation of gene expression of TNF-α, IL-1β, IκBα, NF-κB p65, MCP-1, CXCL1, COX-2, and iNOS in the kidney. Our evidence also suggested that the JPYS formula ameliorates oxidative stress by promoting antioxidant function according to antioxidant index indicators as an indicator of GSH, SOD, CAT, and GPx and abating excessive accumulation of the reactive oxygen species biomarkers, including ROS, TBARS, 8-oxo-dG, and MDA. The data also suggested that the JPYS formula reversed the downregulation of HO-1 and Nrf2 level and upregulation of Keap1 expression. Together, our data highlighted that the JPYS formula relieved renal oxidative injury mediated by activation of Nrf2 signaling by inhibiting inflammation and apoptosis in CKD rats.

## Introduction

Chronic kidney disease (CKD), characterized by the abnormal structure and degenerative dysfunction of the kidney, may cause a series of deleterious influences on people, contributing to increasing morbidity, disability, mortality, and hospitalization expenses worldwide (Webster et al., 2017). Nowadays, despite recent progress in the knowledge on CKD pathogenesis, the treatment is still a challenge and no specific targets and effective therapies are currently available for improving renal function and fibrosis. Renin–angiotensin system inhibitors, the mainstay for treating CKD, are unhelpful to stop its progression toward end-stage renal disease (de Zeeuw, 2011); moreover, they might cause many adverse effects (Budde et al., 2007; Fu-de, 2015). Consequently, several or many researchers are exploring novel target and safe therapeutic strategies to enhance adherence for the prevention and treatment of CKD.

Oxidative stress is in a state of imbalance between oxidants and antioxidants (Daenen et al., 2019), and increasing evidence confirmed that it plays an important role in kidney disease progression (Himmelfarb, 2005) and complications of CKD (Hambali et al., 2011). Nuclear factor erythroid 2–related factor 2 (Nrf2) takes part in maintaining the cellular redox homeostasis by regulating expressions of proteins related to the antioxidative function (Gorrini et al., 2013). In pathogenetic condition, Nrf2 can be activated by the Kelch-like ECH-associated protein 1 (Keap1) in a ubiquitination-dependent manner, contributing to subsequent proteasomal degradation (Itoh et al., 1999). Keap1 will show a conformational change once its cysteine resides, as sensors of oxidative stress, contacted with oxidizers (Dinkova-Kostova et al., 2002), and hamper the combination with newly synthesized Nrf2, which fleetly gathers into the nucleus (Kansanen et al., 2013). As a consequence, Nrf2 can assemble the RNA polymerase machinery and then initiate transcription by combining with small Maf proteins (sMaf) and antioxidant responsive elements (ARE) in the promoters of its target genes (Hirotsu et al., 2012). Many Chinese herbs have been demonstrated to promote the accumulation of Nrf2 and translocation into the nucleus (Zhang et al., 2017) and reinforce gene expression of other antioxidative-related factors (Lu et al., 2019), but whether the possible mechanism of the JPYS formula associates with activation of Nrf2 signaling pathway remains unknown.

Our previous studies and others have highlighted that the JPYS formula exerted an inhibitory effect on the development and progression of CKD according to the rationale of invigorating kidney and strengthening spleen (Wang et al., 2017a; Chen et al., 2019; Zheng et al., 2020), which are considered important roles in the etiology and pathogenesis of renal disease in TCM theory. It has been proved that oxidative stress could result in mitochondrial damage (Ogurtsova et al., 2017) and the JPYS formula could help to restore the aforementioned aspects of the mitochondrial quality control network (Liu et al., 2018). So, we inferred that the JPYS formula might contribute to suppressing oxidative stress. The presence and severity of systemic inflammation contribute to CKD-associated oxidative stress (Himmelfarb et al., 2002; Halliwell, 2007). A previous publication demonstrated the therapeutic effect of the JPYS formula on CKD, including regulating inflammatory cytokines production (Lu et al., 2018). However, the protective effect of the JPYS formula on renal function in connection with inhibiting inflammatory pathways evoked by oxidative stress is still to be elucidated. Comprehensive data with the help of network pharmacology revealed that there are several significant herbs in the JPYS formula of the highest frequency for the treatment of CKD (Xia et al., 2020). Based on previous evidence, the present study aims to comprehensively evaluate the antioxidative effect of the JPYS formula on CKD in 5/6 nephrectomized rats and reveal its chemical constituents and active compounds.

## Materials and Methods

### Composition and Preparation of Jian-Pi-Yi-Shen Formula

The Chinese herbs used in the JPYS formula are presented in Table 1. Raw herbs were purchased from Lingnan Traditional Chinese Pharmaceutical Co., Ltd. (Guangzhou, China), which were identified by Professor Yajun Yang. The voucher specimens were kept at the Department of Pharmacology of Guangdong Medical University. Assurance of quality control for all the materials was validated according to the Chinese Pharmacopoeia (China Pharmacopoeia Committee, 2015). Preparation procedures of the JPYS formula extract were conducted as described in our previous publication (Wang et al., 2017a). In brief, JPYS formula herbs were weighed and extracted in boiling water (1.2 L) twice for 1 h. After centrifugation, the supernatant was dried under reduced pressure to powder, and it was stored at −80°C. Before the treatment, the powder was redissolved with distilled water and vortexed at room temperature to obtain the JPYS extract. The JPYS extract was chemically standardized before administration to animals. An HPLC fingerprint at 260 nm was used as a standard reference for quality control of the JPYS extract (Supplementary Figure S1A,B). An individual reference standard was employed to confirm numerous chemical components, which should be identified from the extract by HPLC analysis, including sodium danshensu, echinacoside, acteoside, calycosin 7-O-β-glucoside, salvianolic acid B, formononetin, and rhein. Besides, the minimal quantitative requirements for echinacoside, salvianolic acid B, and rhein should be no less than 1.2, 5.7, and 0.2 mg/g of the dried extract, respectively. The yield of the extraction was less than 32.59 ± 1.1% (w/w, mean ± SD, *n* = 3). The extract being used here reached the aforesaid requirements.

**TABLE 1:** Chinese herbs of JPYS formula.

Latin name	English name	Chinese name	Palace of origin	Quantities (g)
*Astragalus membranaceus* (Fisch.) Bge. *var. mongholicus* (Bge.) Hsiao	*Astragali Radix*	Huang-Qi	Gansu, China	30
*Atractylodes macrocephala* Koidz.	*Atractylodis Macrocephalae Rhizoma*	Bai-Zhu	Zhejiang, China	10
*Dioscorea opposita* Thunb.	*Dioscoreae Rhizoma*	Shan-Yao	Henan, China	30
*Cistanche deserticola* Y. C. Ma	*Cistanches Herba*	Rou-Cong-Rong	Xinjiang, China	10
*Amomum kravanh* Pierre ex Gagnep.	*Amomi Fructus Rotundus*	Dou-Kou	Guangxi, China	10
*Salvia miltiorrhiza* Bunge	*Salviae Miltiorrhizae Radix et Rhizoma*	Dan-Shen	Shandong, China	15
*Rheum palmatum* L.	*Rhei Radix et Rhizoma*	Da-Huang	Gansu, China	10
*Glycyrrhiza uralensis* Fisch.	*Glycyrrhizae Radix et Rhizoma Praeparata cum Melle*	Zhi-Gan-Cao	Neimengu, China	6

### Animal Experiments

All animal experiments were approved by the Ethics Committee of Guangdong Medical University. Male Sprague-Dawley rats were purchased from Guangdong Medical Laboratory Animal Center (GDMLAC, China), Permission No. SCXK (YUE) 2018–0003, weighing 190–220 g. The animals were housed at room temperature (20 ± 1°C) on a 12:12 h light-dark cycle and had access to water and food ad libitum. CKD was induced in rats by nephrectomy of the right kidney and ablation of two-thirds of the left kidney (5/6 nephrectomy), as we have previously reported (Wang et al., 2014). Sham surgery consisted of anesthetic, flank incision exposing the kidney, and closure of the abdominal wall. All animal experiments were conducted in accordance with the National Institutes of Health (NIH) Guide for the Care and Use of Laboratory Animals and were approved by the Ethics Committee for Experimental Animals of Guangdong Medical University (Approval No.: GDY1902063).

Serum creatinine (SCr) levels were measured 16 weeks after the second surgery and only animals with significantly high SCrlevels were included in the study and randomly divided into four groups: 1) chronic kidney disease group (CKD); 2) low-dose JPYS-treated group (JPYSF-L; 5.4 g/kg/d); 3) medium-dose JPYS-treated group (JPYSF-M; 10.8 g/kg/d); 4) high-dose JPYS-treated group (JPYSF-H; 21.6 g/kg/d). The sham and untreated CKD groups were treated with the same amount of distilled water. The drugs were administered for 8 weeks. All rats used in this study received humane care. During our study, no treatment-related death occurred.

### Serum and Urine Biochemistry Assays

The 24 h urine samples were collected using metabolic cages. Serum biochemical indexes SCr, blood urea nitrogen (BUN), and 24 h urinary protein excretion were measured using commercial kits (Nanjing Jiancheng Bioengineering Institute Nanjing, China) according to the instructions of the manufacturers.

### Histopathology and Immunohistochemistry Detection

Paraffin-embedded rat kidney sections (5 μm thickness) were prepared as a routine procedure. The sections were stained with hematoxylin and eosin (H&E) and Masson’s trichrome staining using standard protocol. The extent of glomerular sclerosis was assessed as described in our previous publication (Wang et al., 2014). Terminal deoxynucleotidyl transferase (TdT)-mediated dUTP nick end labeling (TUNEL) apoptosis detection kit (Promega Corporation, Madison, America) was used to detect apoptosis in accordance with the manufacturer's instructions. Immunoperoxidase staining was performed as described in our previous publication (Wang et al., 2015). Briefly, the sections were deparaffinized by three xylene washes and hydrated by alcohol and washed in distilled water. Three percent hydrogen peroxide was employed to block endogenous peroxidase activity. Antigen retrieval was performed by a microwave oven for 15 min in a citrate buffer (10 mM, pH 6.0). After washing in distilled water and phosphate buffer saline, sections were incubated with 5% BSA at room temperature for 30 min. Then, sections were incubated with primary antibodies against fibronectin (1:100, Ab268020, Abcam, Cambridge, MA, United States), CD68 (1:100, Ab121212, Abcam, Cambridge, MA, United States), and NRF2 (1:100, Ab137550, Abcam, Cambridge, MA, United States) at 4°C overnight. Negative controls excluded the primary antibody. The sections were incubated with a secondary antibody at room temperature for 2 h after being washed with phosphate buffer saline. The reaction was visualized with diaminobenzidine. Counterstaining was performed with 50% Harris hematoxylin. The sections were mounted with neutral gum. The positive area was quantified using ImageJ software (ImageJ 1.32 j, NIH, Bethesda, MD, United States) by limiting the measure to thresholds. The high and low thresholds were assigned by determining the average low and high thresholds for the staining procedure and manually recording the ideal threshold values to distinguish the ubiquitin-positive area. Data were again expressed as percent values of ubiquitin-positive area per total area. Image analysis was done using Image-Pro Plus 6.0 software.

### Determination of Oxidative Stress

ROS levels in the kidney were measured using dihydroethidium (DHE) staining (Beyotime Biotechnology, Shanghai, China). The fluorescence was evaluated in a confocal microscope (Zeiss LSM510Meta). Laser excitation at 488 nm and emission at 610 nm were used. The detection was made using a 560 nm long-pass filter. ImageJ (NIH) software was applied to analyze the fluorescent images quantitatively. The results were shown as arbitrary units of fluorescence (Quick and Dugan, 2001). Thiobarbituric acid reactive species (TBARS) were measured as a lipid peroxidation (LPO) marker (Ohkawa et al., 1979). 8-Oxo-20-deoxyguanosine (8-oxo-dG), an oxidatively modified guanine nucleoside, was detected by an 8-oxo-dG kit (Berry & Associates, Dexter, MI, United States), following the supplied instructions. The MDA contents were measured by chemical colorimetry method as we described previously, using a protein carbonyl colorimetric assay kit (Wang et al., 2018). Reduced glutathione (GSH) (Beutler et al., 1963), the antioxidant enzymes superoxide dismutase (SOD) (Marklund and Marklund, 1974), catalase (CAT) (Cohen et al., 1970), and glutathione peroxidase (GPx) (Matkovics et al., 1998) were tested in the renal tissue homogenates.

### Real-Time RT-PCR Method

mRNA expression was analyzed by quantitative real-time reverse transcription-polymerase chain reaction (RT-PCR) using the real-time PCR thermocycler (Bio-Rad, United States) with SYBR Green PCR Master Mix (Applied Biosystems, Carlsbad, CA, United States), as described in our previous publication (Wang et al., 2020). Real-time RT-PCR was performed in a 20 µL reaction mixture containing 10 μl of SYBR Green Master Mix (Applied Biosystems, Carlsbad, CA, United States), 10 pmol of forward primer, 10 pmol of reverse primer, and 1 μg of cDNA by using a Stratagene Mx3005P QPCR System (La Jolla, CA, United States). Standard procedures were used for quantitative PCR. Primers used in this study are shown in Supplementary Table S1, Supporting Information. The comparative threshold cycle method was used to calculate the mRNA expression of the target genes normalized to glyceraldehyde 3-phosphate dehydrogenase (GAPDH). The 2-ΔΔCt method was used to analyze relative gene expression levels.

### Western Blot Analysis

Aliquots of kidney tissue (40 mg) were snap-frozen and ground in a mortar, thawed, and homogenized in lysis buffer and centrifuged at 15,000 × g for 30 min at 4 °C. Protein concentrations were determined by a Bio-Rad microplate assay, which was a modification of the Bradford assay. Protein bands were scanned and quantified using the ChemiDoc^TM^ MP Imaging System (Bio-Rad Laboratories, CA, United States), as we described previously (Wang et al., 2015). Band densities were normalized by GAPDH levels. Results are expressed as the integrated optical density relative to the corresponding GAPDH value. The following primary antibodies were employed (dilution): Bax (1:100, Ab32508; Abcam, Cambridge, MA, United States), Bcl-2 (1:100, Ab196495; Abcam, Cambridge, MA, United States), Nrf2 (1:1000, #12721; Cell Signaling Technology, United States), Keap1 (1:1000, #4678; Cell Signaling Technology, United States), HO-1 (1:1000, #43966; Cell Signaling Technology, United States), and GAPDH (1:1000, 60004-1-Ig; Proteintech Group, United States).

### Statistical Analysis

All data are expressed as the mean ± standard error of the means (SEM) or standard deviation (SD). SPSS 12.0 K software was used for statistical analysis. One-way ANOVA was used for comparisons among groups. Significant differences between the mean values were assessed by Tukey’s multiple range tests. *p* < 0.05 was considered to be statistically significant.

## Results

### Jian-Pi-Yi-Shen Formula Relieved Impairment of Renal Function in Chronic Kidney Disease Rats

**FIGURE 1:**
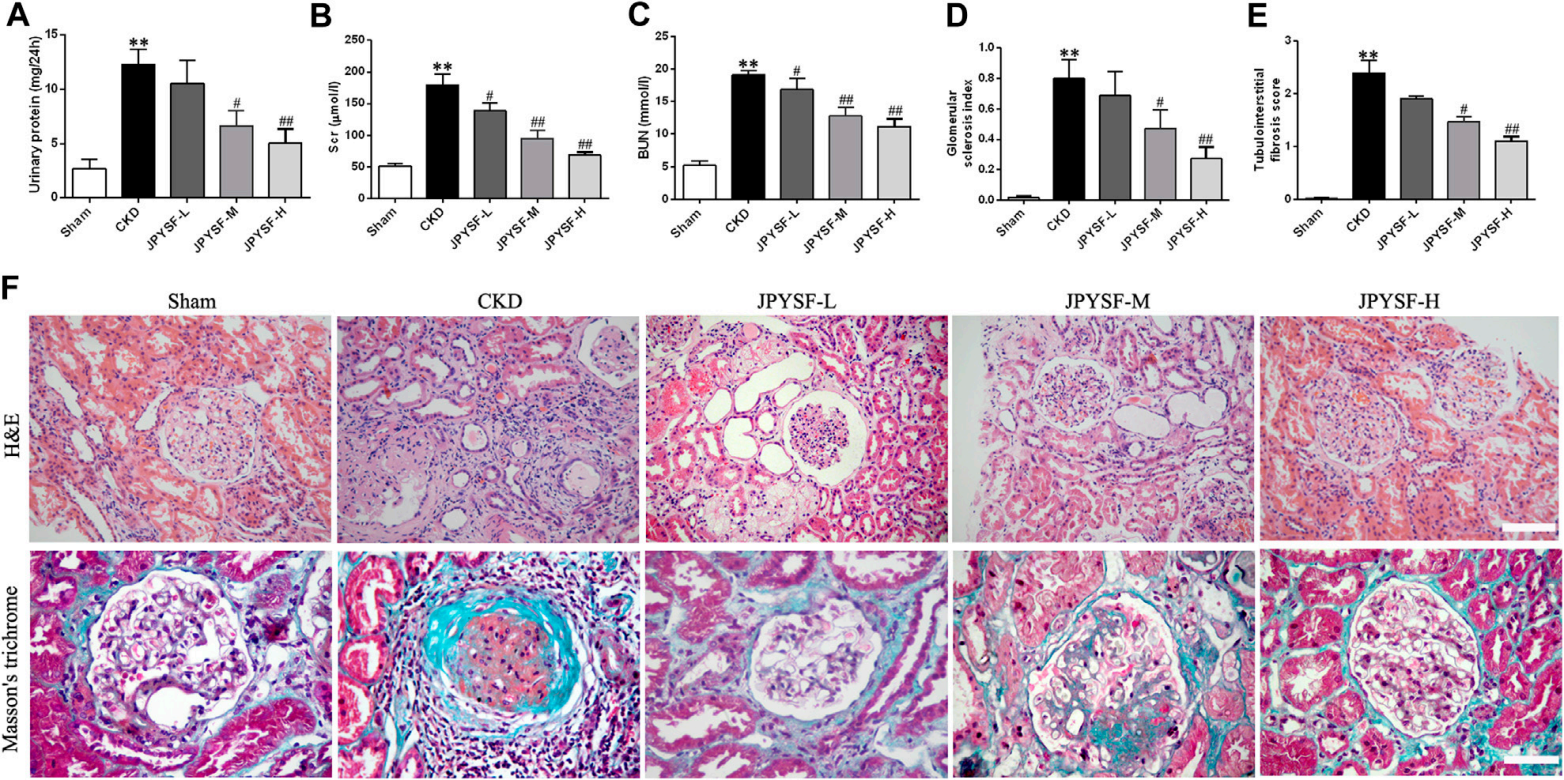
JPYS formula improves renal function and alleviates renal histopathology and ultrastructural pathology of the kidneys from CKD rats. **(A)** 24 h urinary protein excretion. **(B)** Serum creatinine (SCr). **(C)** Blood urea nitrogen (BUN). **(D)** Glomerular sclerosis index. **(E)** Tubulointerstital fibrosis score. **(F)** Hematoxylin and eosin (HE×200, scale bar = 100 μm) and Masson’s trichrome stains (×400, scale bar = 50 μm). All data are expressed as means ± SEM. *p < 0.05, **p < 0.01 vs. the sham group; #*p* < 0.05, ##*p* < 0.05 vs. CKD group.

To evaluate the efficacy of the JPYS formula on the pathological injury of the kidney, a dose-response relationship was firstly ascertained by the biomarkers and ultrastructure related to renal function in the CKD rats. The results showed that impaired renal function of rats in the three groups treated with JPYS formula showed a distinct trend toward reduction in a concentration-dependent manner when compared with the CKD group, based on the correlative parameters, including urinary protein, SCr, BUN, glomerular sclerosis index, and tubulointerstitial fibrosis score, and a significant difference was observed in the high-dose group (Figures 1A–E). Meanwhile, Figure 1F shows images of kidney sections obtained by H&E and Masson’s trichrome staining where the expansion of mesangial area and glomerular hypertrophy were alleviated in the three treatment groups, similar to the effect of the JPYS formula on tubulointerstitial lesions including fibrosis and tubular atrophy, especially in the high-dose group. Based on these results, the high-dose group was adopted together with the sham group and the CKD group in the following experiments.

### Jian-Pi-Yi-Shen Formula Reverse Renal Fibrosis in Chronic Kidney Disease Rats

**FIGURE 2:**
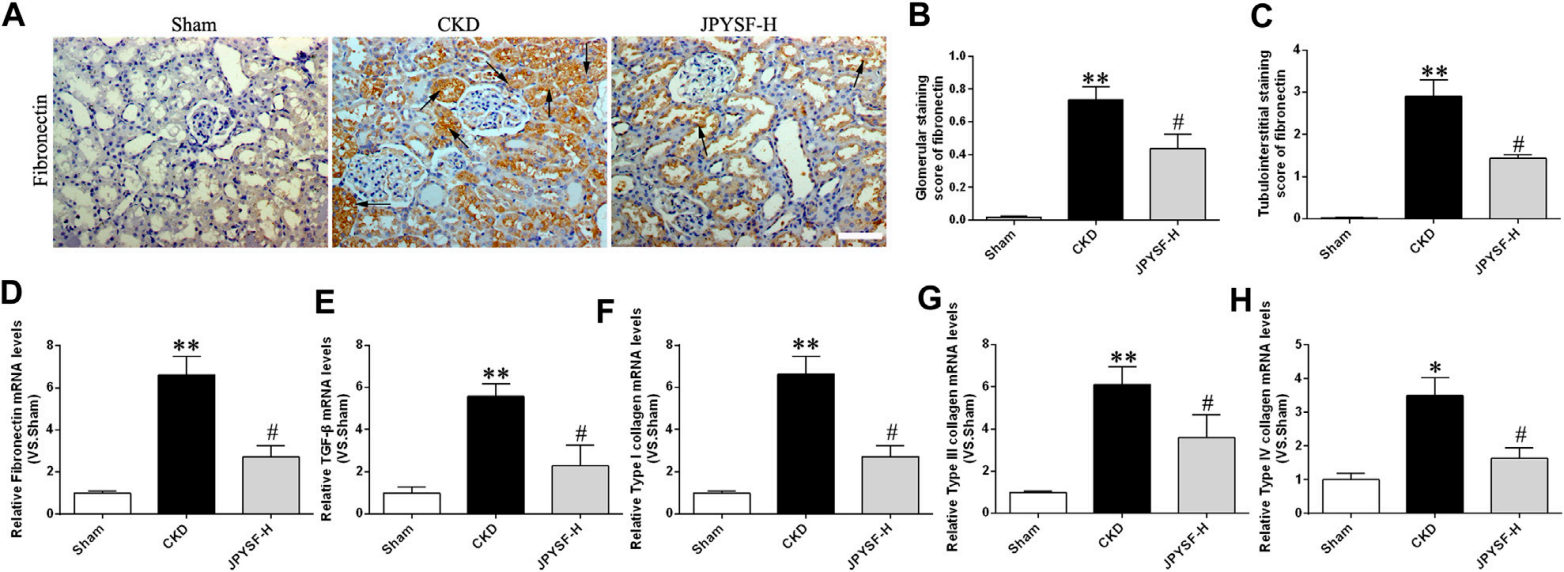
JPYS formula ameliorates renal fibrosis in CKD rats. **(A)** IHC staining of fibronectin in the kidney (×200, scale bar = 100 μm). Fibronectin-positive areas are indicated by arrows. **(B)** Glomerular and **(C)** tubulointerstitial staining score of fibronectin. Relative mRNA expression levels of fibronectin **(D)**, TGF-β **(E)**, type I collagen **(F)**, type III collagen **(G)**, and type IV collagen mRNA **(H)**. All data are expressed as means ± SEM. **p < 0.01 vs. the sham group; #*p* < 0.05 vs. CKD group.

The anti-fibronectin antibody staining of the renal tissue showed that there were large numbers of fibronectin-positive areas in both glomeruli and interstitium of CKD rats, while this change was drastically reduced in the group treated with JPYS formula at high dose (Figures 2A–C). Fibronectin mRNA in the CKD group was also statistically increased compared to the sham group, whereas JPYS formula treatment prevented the effect (Figure 2D). As shown in Figures 2E–H, TGF-β and type I, III, and IV collagen mRNA levels were significantly increased in CKD rats and it was downregulated after treatment with JPYS formula. Collectively, these data suggested that the JPYS formula arrested the development of renal fibrosis responsible for the progression of CKD in 5/6 nephrectomized rats.

### Jian-Pi-Yi-Shen Formula Attenuated Inflammation of Renal Tissue in Chronic Kidney Disease Rats

**FIGURE 3:**
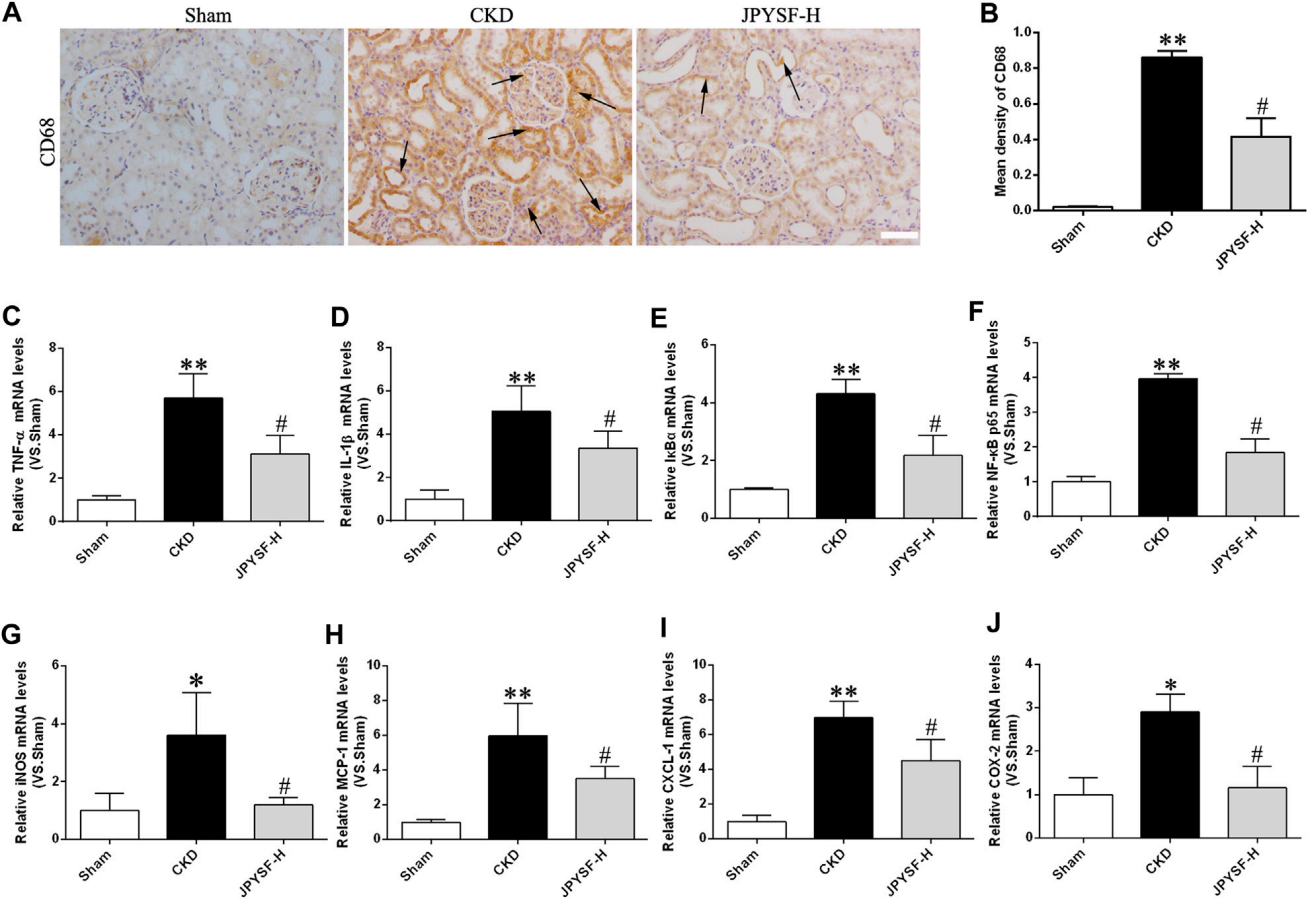
JPYS formula suppresses inflammation in CKD rats. **(A)** IHC staining of CD68 in the kidney (×200, scale bar = 100 μm). CD68-positive areas are indicated by arrows. **(B)** Quantification of CD68 density. Relative mRNA expression levels of TNF-α **(C)**, IL-1β **(D)**, IκBα **(E)**, NF-κB p65 **(F)**, iNOS **(G)**, MCP-1 **(H)**, CXCL-1, **(I)** and COX-2 **(J)**. All data are expressed as means ± SEM. *p < 0.05, **p < 0.01 vs. the sham group; #*p* < 0.05 vs. CKD group.

Persistent, low-grade inflammation is now considered a hallmark feature of CKD. To further explore the anti-inflammatory role of the JPYS formula in CKD pathogenesis, CD68-positive macrophages were observed by immunohistochemistry assay because CD68 is a biomarker of macrophages. The CKD group had much more expression of CD68-positive macrophages in renal interstitium than the sham group, while the JPYS formula improved the overall alteration of morphological features (Figures 3A,B). Additionally, we examined the expressions of proinflammatory cytokines using the qPCR method to further appraise the inflammatory response status of renal tissue in CKD rats. The results showed that the JPYS formula hampered the increased mRNA expression of TNF-α, IL-1β, IκBα, and NF-κB p65 and its target gene iNOS ( Figures 3C–G). Furthermore, the increased mRNA levels of targets of NF-κB were suppressed by the JPYS formula, including MCP-1, CXCL-1, and COX-2 (Figures 3H–J). The results indicated a significant inhibitory effect of JPYS formula on CKD-aroused inflammatory response via downregulation of activation of NF-κB pathway in the kidneys of the treated rats. Taken together, these lines of evidence demonstrated that the JPYS formula showed anti-inflammatory effects and the underlying mechanism in CKD rats.

### Jian-Pi-Yi-Shen Formula Inhibited Oxidative Stress and Enhanced Antioxidant Capacity in Chronic Kidney Disease Rats

**FIGURE 4:**
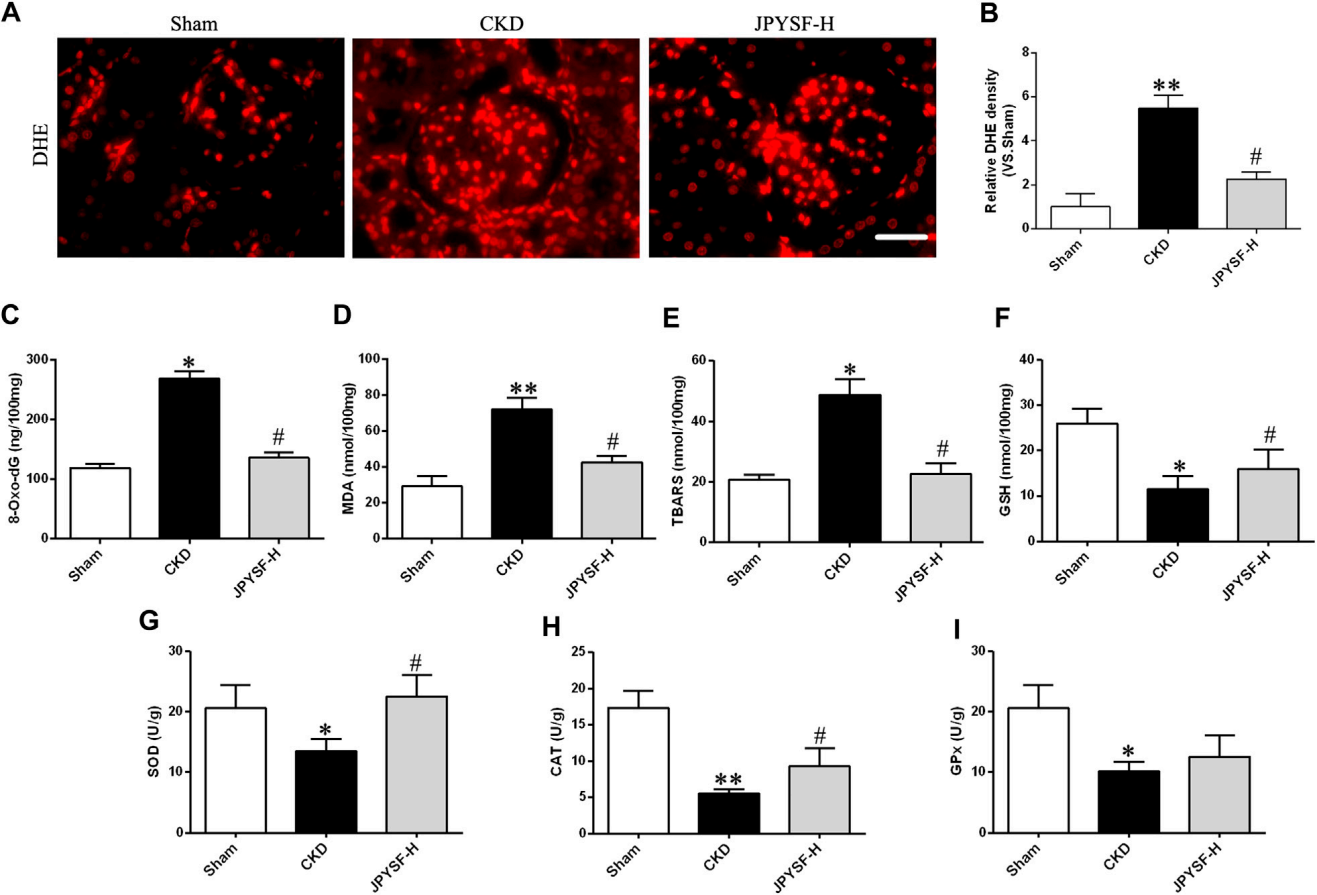
JPYS formula suppresses oxidative stress in the kidneys from CKD rats. **(A)** Immunofluorescence staining for dihydroethidium (DHE) in the kidney (×400, scale bar = 50 μm). **(B)** Quantification of DHE intensity. Relative expression levels of 8-oxo-dG **(C)**, MDA **(D)**, TBARS **(E)**, GSH **(F)**, SOD **(G)**, CAT **(H),** and GPx **(I)**. All data are expressed as means ± SEM. *p < 0.05, **p < 0.01 vs. the sham group; #*p* < 0.05 vs. CKD group.

The imbalance of redox status plays a vital role in intracellular signaling during the pathogenic process of CKD. To evaluate the oxidative stress level, the generation of ROS was measured by dihydroethidium (DHE) staining in renal tissue, and the other indexes in response to excessive ROS accumulation were determined in the serum samples of CKD rats. As shown in DHE staining and its intensity quantification (Figures 4A,B), the JPYS formula displayed a significant inhibitory influence on the increased ROS level of renal tissue in CKD rats and serum 8-oxo-dG, a key biomarker of oxidative damage to cellular DNA (Figure 4C). Similar effects of the JPYS formula on lipid peroxidation were confirmed by the results acquired from the measurements of MDA and TBARS (Figures 4D,E). On the other hand, the JPYS formula boosted renal antioxidant defenses through activation enzymes with ROS scavenging activities, including GSH, SOD, CAT, and GPx (Figures 4F–K). Taken together, these data revealed that the JPYS formula may exert a beneficial effect on redox balance via reduction of oxidative status and enhancement of antioxidant capacity, contributing to abatement of the oxidative stress–induced deleterious effect on renal tissue in the pathogenesis of CKD.

### Jian-Pi-Yi-Shen Formula Arrested Caspase-Dependent Apoptosis in Chronic Kidney Disease Rats

**FIGURE 5:**
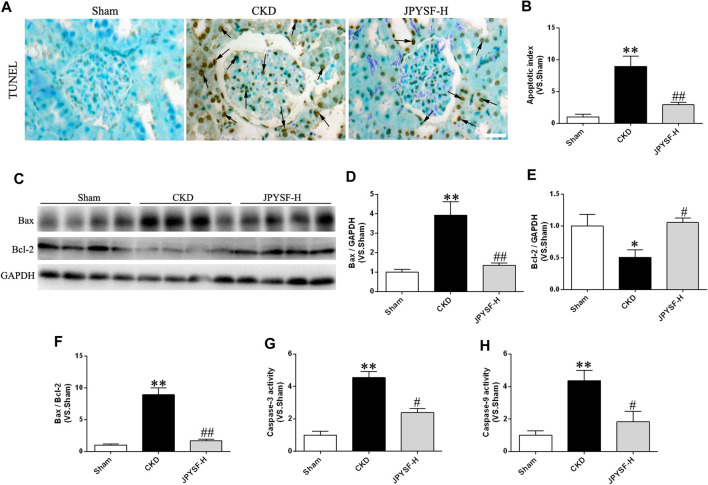
JPYS formula prevents renal cell apoptosis in CKD rats. **(A)** Terminal dUTP nick end labeling (TUNEL) staining in the kidney (×400, scale bar = 50 μm). TUNEL-positive nuclei are indicated by arrows. **(B)**The number of TUNEL-positive cells is expressed as the percentage of total cells. **(C)** Representative western blots for Bax, Bcl-2, and GAPDH protein expression in kidney tissue. **(D)** Quantitative analysis of Bax/GAPDH. **(E)** Quantitative analysis of Bcl-2/GAPDH. **(F)** Quantitative analysis of Bax/Bcl-2. The activity of caspase-3 **(G)** and caspase-9 **(H)**. All data are expressed as means ± SEM. *p < 0.05, **p < 0.01 vs. the sham group; #*p* < 0.05, ##*p* < 0.05 vs. CKD group.

It is well known that oxidative damage and inflammation may be especially vulnerable to driving cell death via apoptosis in a caspase-dependent manner. Given that the pathogenic process of CKD was associated with oxidative stress and inflammation, it was necessary to explore the effect of the JPYS formula on apoptosis. As illustrated in Figures 5A,B, the JPYS formula ameliorated the apoptotic rate according to the result of TUNEL staining and intensity quantification. Meanwhile, the JPYS formula counteracted CKD-mediated apoptosis evidenced by the increased protein abundance of Bax with a concomitant decline in Bcl-2 expression along with an increased Bax/Bcl-2 ratio (Figures 5C–F). Moreover, the JPYS formula hampered the CKD-triggered increased activity of caspase-3, located downstream of caspase-9 in the apoptotic signaling pathway, and significantly attenuated the activity of caspase-9 (Figures 5G,H). The findings provide further support that the JPYS formula may lead to activation of caspase-9 via downregulation of Bax and upregulation of Bcl-2, contributing to activation of caspase-3, and arrested cellular apoptosis ultimately in the renal tissue of CKD rats.

### Jian-Pi-Yi-Shen Formula Hampered Downregulation of Nuclear Factor Erythroid 2–Related Factor 2 Signaling Elicited by Chronic Kidney Disease

**FIGURE 6:**
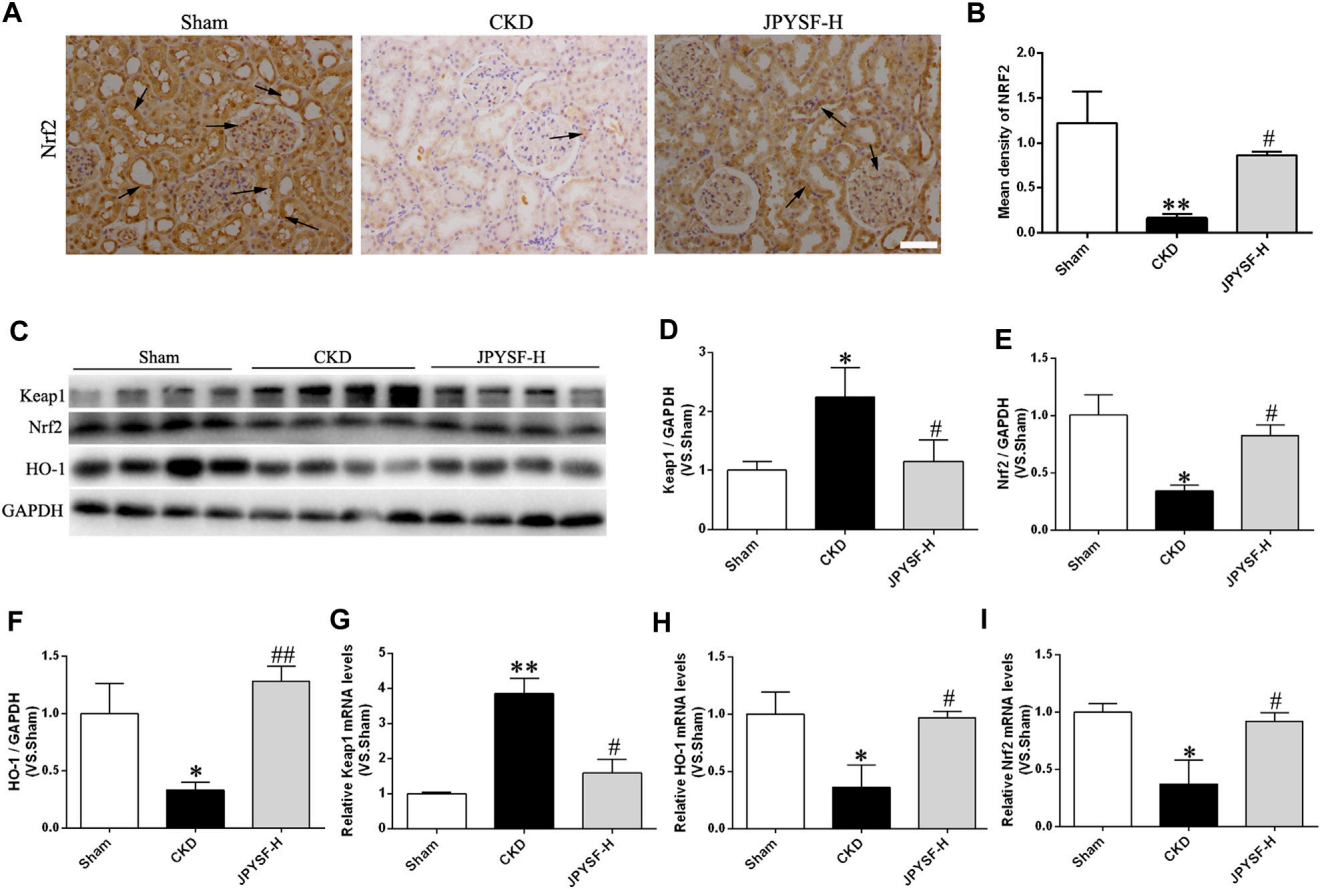
JPYS formula activates the Nrf2 signaling in the kidneys from CKD rats. **(A)** IHC staining of Nrf2 in the kidney (×200, scale bar = 100 μm). **(B)** Quantification ofNrf2 density. Nrf2-positive areas are indicated by arrows. **(C)** Representative western blots for Keap1, Nrf2, HO-1, and GAPDH protein expression in kidney tissue. **(D)** Quantitative analysis of Keap1/GAPDH. **(E)** Quantitative analysis of Nrf2/GAPDH. **(F)** Quantitative analysis of HO-1/GAPDH. Relative mRNA expression levels of Keap1 **(G)**, HO-1 **(H)**, and Nrf2 **(I)**. All data are expressed as means ± SEM. *p < 0.05, **p < 0.01 vs. the sham group; #*p* < 0.05, ##*p* < 0.05 vs. CKD group.

Concerning the evidence that Nrf2 is taken as an inactive complex in the cytoplasm by the repressor molecule Keap1, which facilitates the ubiquitination of Nrf2, we next asked whether the cellular antioxidant capacity improved by JPYS formula via activation of Nrf2/HO-1 signaling pathway. The evidence acquired from the results of immunohistochemical staining, Western blot, and qRT-PCR assay proved that the JPYS formula reversed the decreased expression of Nrf2 and its target gene so-called HO-1 in CKD rats and the increased level of Keap1 (Figures 6A–I). In brief, the preventive role of the JPYS formula on the redox balance of renal tissue in CKD rats may involve the activation of the Nrf2/HO-1 signaling pathway.

## Discussion

Oxidative stress triggered by excessive ROS production has been found to be associated with the initiation and progression of CKD. Continued oxidative stress can elicit chronic inflammation, which can be exemplified in the process of renal injury and fibrosis, ultimately contributing to kidney dysfunction. In accordance with the report on renoprotection of JPYS formula on anti-inflammation (Chen et al., 2018), we found that JPYS formula relieved inflammatory injury in CKD mice by inhibiting the activation of NF-κB pathway via loss in the kidney. It was reported to reduce the cell apoptosis on oxidative impairment of renal tubular epithelial cells of rats (Dan, 2015) and induce the accumulation of hypoxia-inducible factor-α (HIF-α) protein expression in CKD anemic rats (Chen et al., 2019). Here, we confirmed the protective effect of the JPYS formula on renal tissue through enhancing antioxidative capacity and arresting apoptosis in a caspase-dependent manner. Similarly, we demonstrated that the JPYS formula exerted a therapeutic effect of improving renal dysfunction according to urinary protein and SCr excretion and fibrosis status based on improving the parameters related to the profibrogenic factors, such as fibronectin, TGF-β, and collagen I, III, and IV. Particularly, we highlighted the preventive role of JPYS formula in kidney dysfunction in the light of maintaining redox balance via activation of Nrf2/HO-1 signaling pathway in CKD rats.

Oxidative stress inevitably occurred in various models of CKD. Rats with 5/6 nephrectomy-induced CKD show severe oxidative stress, increased activity of oxidases and the subsequent ROS accumulation, strong inflammatory response, and activated NF-κB pathway activation in the remnant kidney (Fujihara et al., 2007; Cho et al., 2009). Renal functional and morphological features can be ameliorated by antioxidant treatment (Shah et al., 2007), by which a novel approach may be developed for treating CKD. *Dioscoreae Rhizoma* and *Cistanches Herba* as main ingredients in the JPYS formula have been shown to have strong free radical scavenging capacity (Fu et al., 2018) and protective effects on homeostasis as antioxidants (Wang et al., 2017b; Qiao et al., 2018). In this study, JPYS formula can make impaired kidney restore antioxidative function to some extent in CKD rats. Excessive ROS production is remarkably damaging to DNA, protein, and lipid, exceeding the capacity of the natural antioxidant defense system via attacking, denaturing, and modifying renal structural and functional molecules, causing kidney tissue damage and dysfunction. Oxidative stress is driven by impaired antioxidant defense or excessive ROS production, which is primarily induced by activation and increase of ROS-producing enzymes, including mitochondrial dysfunction (Malhotra and Kaufman, 2007; Cachofeiro et al., 2008). Our previous study has proved that the JPYS formula (Liu et al., 2018) could restore the aforesaid aspects of the mitochondrial quality control network, which might be that the JPYS formula method abates the ROS generation. In pathological conditions, the enzymatic antioxidants will be mobilized to clear or metabolize oxidate substances, which maintain a lessening tone within cells and avoid harmful oxidative conditions (Daenen et al., 2019). Our study showed that the JPYS formula can counteract ROS accumulation and oxidative stress and strengthen activities of the enzymatic antioxidants such as SOD, CAT, and GSH, illustrating that the JPYS formula could restore redox balance by activating the antioxidant defense systems.

It well known that Nrf2 is considered a master regulator of genes in response to antioxidative stress due to encoding many antioxidant and detoxifying enzymes (Nezu and Suzuki, 2020). Impaired activation of Nrf2 attenuates or abrogates the response of encoding antioxidants genes, and Nrf2 KO rats exhibit a much more severe renal dysfunction and structural deformation, contributing to many CKDs, such as lupus-like autoimmune nephritis and diabetic nephropathy induced by oxidative stress (Yoh et al., 2001). Therefore, there is a need to elucidate how the JPYS formula protects renal function from oxidative injury by regulating the Nrf2 pathway in CKD rat. However, the main active component of the JPYS formula has been proved to have antioxidant activity by increasing the expression of cellular GSH by activating the Nrf2 gene (Adesso et al., 2018). Under quiescent conditions, Keap1, a direct binding molecule to Nrf2, represses Nrf2 transactivation and retains Nrf2 in the cytoplasm. The cooperative interactions provide overall stability of Nrf2-regulated genes in low basal expression. However, Nrf2 is poised to be released from Keap1 upon sensing stress induced by oxidative and electrophilic molecules and then is translocated into the cell nucleus along with transactivating the expression of related cytoprotective genes, allowing for a prompt protective response and enhancing cell survival. Thus, increased Nrf2 nuclear translocation indicates Nrf2 activation (Suzuki and Yamamoto, 2015). Our present data showed that the JPYS formula caused an upregulation of Nrf2 and HO-1 expression and a decrease of Keap1 level, reflecting that the JPYS formula may contribute to restraining the combination between Nrf2 with Keap1, resulting in failure of proteolysis of Nrf2, and then provokes the activation of antioxidant defense systems in CKD rats. That might be the underlying mechanism of the JPYS formula for treating CKD.

Generally, the inflammatory status of renal cells is constantly changing along with the level of oxidative stress during the pathogenic process of CKD tissues. This study showed two interlinked processes due to the evidence between the extent of infiltration and redox state of kidney injury. Inflammatory cells provoke numerous ROS at the inflammatory capture, resulting in exaggerated oxidative stress, and excessive ROS further promotes initiating intracellular signaling cascade, enhancing the expression of proinflammatory factors (Anderson et al., 1994). The NF-κB activation plays an important role in the inflammatory process, and our data showed that such an increase of IκBα mRNA level could be inhibited by the JPYS formula in CKD rats. Pharmacological and genetic studies indicated a functional cross-talk between Nrf2 and NF-κB pathways. It was reported that Nrf2 KO can exacerbate NF-κB activity, inducing increased inflammatory cytokine (Morimitsu et al., 2002; Chen et al., 2006), and that increases in HO-1 activity in endothelial cells suppress the NF-κB-mediated transcription (Soares et al., 2004). On the other hand, NF-κB activity also regulates Nrf2-mediated ARE expression. NF-κBp65 stifles Nrf2 transcription by deacetylation of histones (Liu et al., 2008) and prevents heterodimer formation with Nrf2; moreover, it was of great assistance to enhance the abundance of nuclear Keap1 (Yu et al., 2011), therefore leading to diminishing Nrf2-ARE–related genes. Collectively, the regulation of Nrf2 in response to NF-κB activation is proved as a protection system against the consequences of inflammation (Cuadrado et al., 2014). In the present study, JPYS improved the insufficiency of Nrf2 and its downstream targets in CKD rats and mitigated infiltration of inflammatory cells, suggesting that the JPYS formula also has an anti-inflammatory effect by activating Nrf2 signaling through suppressing the NF-κB pathway.

## Conclusion

Our study proved that the JPYS formula upregulated the antioxidant response and attenuated proinflammatory signaling, revealing a significant anti-inflammatory and antioxidative effect as evidenced by the activation of Nrf2 signaling. However, our detected biomarkers were not adequate, and the data of antioxidative effect could not directly explain the causal relationship between these changes. Further experiments are needed, such as adding Nrf2 agonists or inhibitors or the knockout model of Nrf2 rats, to go deeper into the role of the Nrf2 signaling pathway in the protection of JPYS formula on CKD rats.

## Data Availability

The raw data supporting the conclusions of this article will be made available by the authors without undue reservation.
