# Supplementary figures and images for: Jian-Pi-Yi-Shen Formula Ameliorates Oxidative Stress, Inflammation, and Apoptosis by Activating the Nrf2 Signaling in 5/6 Nephrectomized Rats
Source: Front Pharmacol. 2021 Mar 25;12:630210. doi: 10.3389/fphar.2021.630210 (PMC8027107; doi:10.3389/fphar.2021.630210)

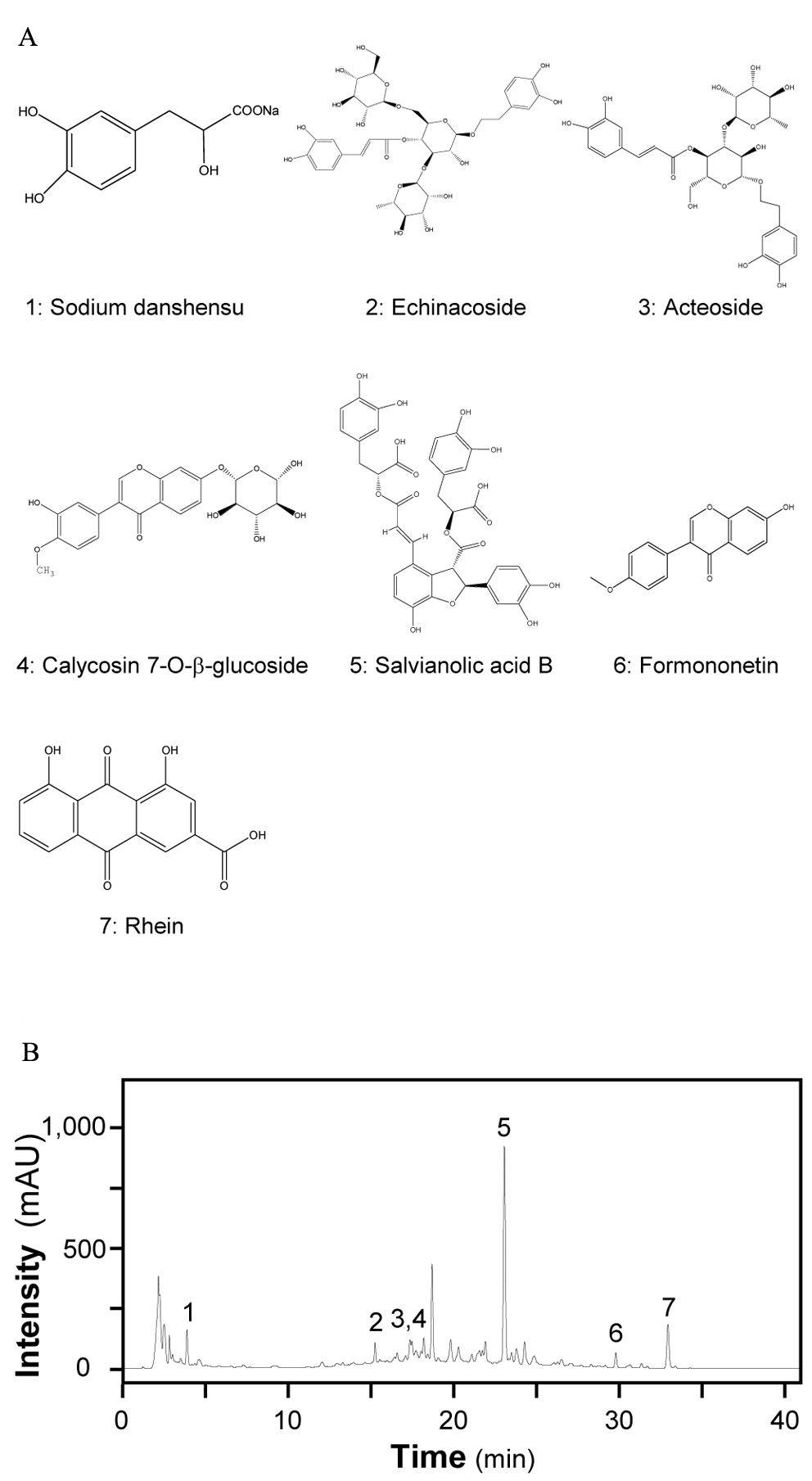

Supplement: Supplementary file 1 [file image1.tif]
