# Supplementary material for: Jian-Pi-Yi-Shen Formula Ameliorates Oxidative Stress, Inflammation, and Apoptosis by Activating the Nrf2 Signaling in 5/6 Nephrectomized Rats
Source: Front Pharmacol. 2021 Mar 25;12:630210. doi: 10.3389/fphar.2021.630210 (PMC8027107; doi:10.3389/fphar.2021.630210)
Supplement: Supplementary file 2 [file table1.docx]

**Table.S1.** Primer sequences for qRT-PCR

| Gene | Forward | Reverse |
| --- | --- | --- |
| Fibronectin | 5′-GTGATCTACGAGGGACAGC-3′ | 5′-GCTGGTGGTGAAGTCAAAG-3′ |
| TGF-β1 | 5′-GCCAGATCCTGTCCAAACTAA-3′ | 5′-TTGTTGCGGTCCACCATTA-3′ |
| Collagen I | 5′-ATCCTGCCGATGTCGCTAT-3′ | 5′-CCACAAGCGTGCTGTAGGT-3′ |
| Collagen III | 5′-CTGGTCCTGTTGGTCCATCT-3′ | 5′-ACCTTTGTCACCTCGTGGAC-3′ |
| Collagen IV | 5′-GCCCTACGTTAGCAGATGTACC-3′ | 5′-TATAAATGGACTGGCTCGGAAT-3′ |
| TNF-α | 5′-GTCTGTGCCTCAGCCTCTTC-3′ | 5′-TGGAACTGATGAGAGGGAGC-3′ |
| MCP-1 | 5′-TCACCTGCTGCTACTCATTC-3′ | 5′-AGTGGATGCATTAGCTTCAG-3′ |
| CXCL-1 | 5′-GGCAGGGATTCACTTCAAGA-3′ | 5′-ACTTGGGGACACCCTTTAGC-3′ |
| IκBα | 5′-CGTGTCTGCACCTAGCCTCTATC-3′ | 5′-GCGAAACCAGGTCAGGATTC-3′ |
| NF-κB | 5′-ACATCCCTCAGCACCATCAA-3′ | 5′-TTGGTACCATGGCTGAGGAG-3′ |
| lL-1β | 5′-TGAAGCAGCTATGGCAACTG-3′ | 5′-CTGCCTTCCTGAAGCTCTTG-3′ |
| COX-2 | 5′-TAC AAC AAC TCC ATC CTC CTT G-3′ | 5′-TTC ATC TCT CTG CTC TGG TCA A-3′ |
| iNOS | 5′-CCAACAACACAGGATGACC-3′ | 5′-CCTGATGTTGCCACTGTTAG-3′ |
| Keap1 | 5′-CCTGTCTGTTGTCTCTGCTTAC-3′ | 5′‐GAAGTTGGGTCATTGGCTTCTA-3′ |
| HO-1 | 5'-ACAGAAGAGGCTAAGACCG-3' | 5'-CAGGCATCTCCTTCCATT-3' |
| Nrf2 | 5′-GCAACTCCAGAAGGAACAGG-3′ | 5′‐AGGCATCTTGTTTGGGAATG-3′ |
| GAPDH | 5'-ACTCTCTTCTTCCCCCTTGC-3' | 5'-TCCACGACATACTCAGCAC-3' |
